# Sociodemographic characteristics of 96 Indian surrogates: Are they disadvantaged compared with the general population?

**DOI:** 10.1371/journal.pone.0214097

**Published:** 2019-03-25

**Authors:** Virginie Rozée, Sayeed Unisa, Elise de La Rochebrochard

**Affiliations:** 1 Sexual and Reproductive Health and Rights Research Unit, Institut National d’Études Démographiques (INED), Paris, France; 2 Department of Mathematical Demography & Statistics, International Institute for Population Sciences (IIPS), Mumbai, India; 3 Université Paris-Saclay, Univ. Paris-Sud, UVSQ, CESP, INSERM, Kremlin-Bicêtre, France; University of Botswana, BOTSWANA

## Abstract

Commercial surrogacy in emerging countries such as India is often associated with exploitation of vulnerable women, the assumption being that it is performed by poor and uneducated women for rich intended parents. However, the hypothesis that surrogates are poor women has rarely been confronted with field data. The objective was to compare the socioeconomic characteristics of Indian surrogates interviewed in social studies with those of Indian women in the general population in order to provide preliminary data on whether surrogates have a specific profile and are indeed disadvantaged compared with their counterparts. The study analyzes the data from four cross-sectional studies carried out in India among surrogates between 2006 and 2014. Surrogates were recruited through clinics, agencies and agents. Data were collected during face-to-face interviews. The resulting convenience sample included 96 Indian surrogates. Their sociodemographic characteristics were compared with those of the general population extracted from Indian national surveys. The surrogates interviewed had their first child at a younger age than women in the general population, but they tended to have a smaller family. Their social situation tended to be better than that of the general population in terms of education, employment and family income. These results provide first empirical evidence moderating the common assumption that Indian surrogates are the poorest and least educated women. This does not mean, however, that exploitation does not exist. More studies are needed to confirm these results and to explore the issue in new international destinations for surrogacy.

## Introduction

Among assisted reproductive technologies (ARTs), surrogacy is a statistically negligible phenomenon as it represents less than 1% of ART procedures in countries such as the United States, which is known to be a key destination for surrogacy [1]. However, this practice has polarized public attention and led to heated social debates [2].

Transnational commercial surrogacy performed in less developed and emerging countries (such as India, Thailand, Nepal and Mexico) is a particularly controversial topic of social debate [3–6]. The main risk under consideration is the risk of exploitation of poor women [7, 8]. Transnational commercial surrogacy in emerging countries has been described as “renting wombs” or “baby trade”, and has sometimes been compared to prostitution.

Since the 2000s, India has become one of the top international destinations for surrogacy [9]. In 2012, it was estimated that more than 25,000 children had been born through surrogacy in India, half of them for international parents [10]. In this country, risk of exploitation is a key concern as poverty is a major national challenge, with 42% of Indian citizens living with less than 1.25 USD per day and 76% with less than 2 USD per day [11]. The risk of exploitation of Indian surrogates has been largely broadcast worldwide in the media. Facing public exposure to controversies and scandals, the Indian government has developed successive ART draft bills since 2008 [12, 13], recommending for example that surrogates should be 21–35 years old, have at least one child and less than five live births.

The main argument developed against Indian surrogacy is that it is a “survival strategy” [14] for the poorest and least educated women in the Indian population [3, 15–17]. Through this perspective, only desperately poor women in need of quick money become surrogates under the assumption that “*no one would choose to sell sex*, *or to rent their wombs*, *if there were any other economic options*” [3]. However, this hypothesis of the extreme poverty of Indian surrogates has rarely been confronted with empirical reality through field data. Numerous papers have been published on surrogacy in India but only a few studies have interviewed Indian surrogates [18–23]. These few ethnographic studies show that the reality of surrogacy in India is more complex than the survival strategy approach usually proclaimed. Nevertheless, these studies relied on small samples, and only a very few describe the socioeconomic profiles of the Indian surrogates interviewed. Thus, they do not enable an approach to the socioeconomic situation of the surrogates compared with the general Indian population. There is still therefore a need to investigate whether surrogates really are at the bottom of the socioeconomic ladder, as is presumed by the argument concerning the exploitation of vulnerable women.

Our objective was to compare the socioeconomic characteristics of Indian surrogates with those of Indian women in the general population in order to determine whether surrogates have a specific profile and are indeed disadvantaged compared with their counterparts.

## Materials and methods

### Studies on Indian surrogates

In the absence of a surrogates’ register and of a list of medical centers and agencies offering surrogacy in India, it is impossible to draw a random sample of Indian surrogates. Investigating surrogacy is all the more difficult given the very large number of clinics and agencies involved, estimated at around 3,000 [21, 24]. The few existing studies are based on a limited number of surrogates recruited through clinics and agencies willing to collaborate in the survey. Such survey methodology is known as convenience sampling as the resulting sample of surrogates cannot be considered as a random sample [25].

We selected all published field studies (n = 4) that provided detailed data on the socioeconomic characteristics of each surrogate, including one study conducted by the authors of this paper [9, 26]. The four field studies used shared the same methodology to recruit the surrogates. These studies are presented in detail elsewhere [9, 14, 19, 21, 26, 27] and are briefly presented below.

All four field studies were conducted by a woman social scientist. They were based on collaboration with medical centers and agencies to obtain the authorization to interview surrogates. Surrogacy is under strict supervision by medical doctors and surrogates are not allowed to share their experience without the express consent of the medical center [21, 22]. Moreover, surrogacy is a taboo and confidential practice and surrogates usually refuse to disclose their experience outside the clinic. The studies were therefore conducted among surrogates with the collaboration of clinics and agencies. All surrogates staying or consulting at the clinic or agency were eligible to participate when the social scientist was *in situ*.

The surrogates were recruited between 2006 and 2014 in six Indian states: 42 surrogates in Guajarat (Anand) in 2006–2008 [27], 12 in Punjab and Delhi in 2011–2012 [21], 9 in West Bengal (Kolkata) in 2012 [19] and 33 in Maharashtra (Mumbai), Tamil Nadu (Chennai) and Delhi (New Delhi) in 2013–2014 [26]. The total convenience sample comprised 96 surrogates (see database in S1 Appendix).

Interviews were directly conducted by the social scientist in local languages and/or with the help of a local translator. Anonymity and confidentiality were ensured and oral free informed consent was obtained. For our own field study carried out in 2013–2014, we received approval from the Ethics Review Board of the Indian IIPS (n°IIPS/ERB/217/2013).

### Indian national sources

Data on Indian women of reproductive age were extracted from the Indian National Family Health Survey [28]. To measure the economic characteristics of the Indian population, data were extracted from the World Development Report [11]. In this report, the international poverty line is defined as 2 USD a day and the extreme poverty line as 1.25 USD a day.

### Statistical analyses

Statistical tests were performed with permutation tests for non-random samples [29, 30], using R software (version 3.4.3, R Foundation for Statistical Computing). Tests were done with the R function “Permutation Tests for Nonparametric Statistics” (perm.test, version 1.4, package: jmuOutlier) developed by Steven T. Garren [31].

## Results

The socioeconomic characteristics of surrogates are shown in Table 1. The 96 surrogates were recruited in the four main Indian regions: Northern (Delhi, Punjab, n = 14), Western (Anand, Mumbai, n = 58), Southern (Chennai, n = 15), and Eastern (Kolkata, n = 9). The majority were aged 21 to 35 years (n = 82/93, 91%) and none was younger than 20 years. Nearly all were living with their husband (n = 85/96, 84%). They mostly had their first child by the age of 20 (n = 33/39, 85%) and they generally had one or two children (n = 73/95, 77%). About half the surrogates had received at least secondary education (n = 51/91, 56%) and had been employed before surrogacy (n = 55/95, 58%). The surrogates’ income was predominantly (n = 47/66, 71%) above the poverty line (2 USD a day).

**Table 1:** Socioeconomic characteristics of 96 Indian surrogates and of Indian women aged 20–34 years old in the general population.

		Indian surrogates (N = 96)		General population of Indian women aged 20–34 (N = 60,852)	P-value of permutation test^(1)^
		n/N	%	%	
**Age**	**(years)**				
	20–24	12/93	13	37	<0.001
	25–29	41/93	44	34	<0.05
	≥30 ^(2)^	40/93	43	29	<0.01
**Indian region**					
	Northern	14/96	15	37	<0.001
	Western	58/96	60	32	<0.001
	Southern	15/96	16	13	0.48
	Eastern	9/96	9	18	<0.001
**Married living with husband**		81/96	84	85	0.87
**Number of children**					
	None	0/95	0	20	NA^(3)^
	1–2	73/95	77	44	<0.001
	3–4	21/95	22	27	0.25
	5	1/95	1	9	<0.001
**First cild at age ≤ 20**		33/39	85	48	<0.001
**At least 2ndary education** ^(4)^		51/91	56	39	<0.01
**Family income ≥ extreme poverty line (1.25 USD)**		56/66	85	58	<0.001
**Family income ≥ poverty line (2 USD)**		47/66	71	24	<0.001
**Employed** ^(5)^		55/95	58	42	<0.01
**Hindu religion**		60/85	71	82	0.02

(1) Permutation tests compared percentages observed among surrogates and in national data for one category. Tests were done with the R function “Permutation Tests for Nonparametric Statistics” (perm.test, version 1.4, package: jmuOutlier) developed by Steven T. Garren.

(2) Age was divided into 5-year groups in order to be comparable with national data. Regarding guidelines for surrogacy, 82 surrogates were aged 21–35 years old, 1 surrogate was aged 20 years and 9 were aged 36–45 (of whom only 2 women were aged 40 years or above).

(3) NA: Not applicable, as no surrogate had 0 children

(4) At least 7 or 8 years of education (secondary, graduate and post-graduate).

(5) Women employed before surrogacy. In the national data, women employed at some point in the 12 months preceding the survey.

The characteristics of the 96 surrogates were compared with those of the general population of Indian women aged 20–34 using permutation tests for nonparametric statistics (Table 1). The surrogates were distinguished from the general population of Indian women aged 20–34 by a higher level of education (56% versus 39%, p<0.01) and greater involvement in the economic market as measured by their occupational status (58% versus 42%, p<0.01). While 85% of surrogates interviewed declared a family income above the international extreme poverty line, only 58% of the Indian general population declared such an income (p<0.001). Using a less strict indicator, the international poverty line, 71% of surrogates declared a family income above 2 USD/day, compared with 24% in the general population (p*<*0.001). All these outcomes, education, occupation and family income, coherently suggest that the surrogates interviewed had a more favourable social situation than the average general population.

The fertility history of surrogates also diverged from that of the general population of Indian women aged 20–34 on two points. Firstly, surrogates tended to become mothers at an earlier age (before age 20) (85% versus 48%, p<0.001), and secondly, they tended to have a smaller family with one or two children (77% versus 44%, p<0.001).

## Discussion

Based on a convenience sample of 96 surrogates, we were able for the first time to explore the socioeconomic characteristics of a sample of Indian surrogates and to compare them with the general population of Indian women. Although limitations due to a non-random sample should be borne in mind, for the first time our results made it possible to go beyond the usual prejudice and negative stereotypes about the profiles of Indian surrogates and to provide the first indications regarding their actual age, reproductive career and socioeconomic status.

The majority of surrogates in our convenience sample were aged 25 years and over. The low proportion of surrogates aged 20–24 years probably reflects the fact that according to the ART draft bill women are required to be mothers before committing to surrogacy. Moreover, in line with the bill’s recommendations, they were mostly (previously) married, with at least one child and less than five children. This finding suggests that national legal and medical guidelines regarding age and family situation were adhered to for the recruitment of surrogates. Indian guidelines on surrogates’ profile are stricter than those applied in other countries. For example, UK regulations allow childless and unmarried women to be surrogates [32].

Among the 96 surrogates interviewed, the proportion of women who had their first child before age 20 was higher than in the general population. However, they tended to have a smaller family, with 77% having one or two children. So, the 96 surrogates were usually below the requirements of the Indian ART Bill that recommends surrogates to have less than five children. Possibly, surrogates may be fertile women who manage to have less children in marriage, for example by undergoing permanent sterilization as observed in one study [20]. However, this does not necessarily mean that surrogates have reproductive autonomy or related decision-making power, as was demonstrated in a study conducted in Tamil Nadu on the complex relationship between fertility decline and female autonomy [33].

Looking at the socioeconomic situation of the 96 surrogates, three dimensions were explored: education, occupation and family income. On all these indicators, the surrogates interviewed consistently and coherently had more favorable socioeconomic status than Indian women in the general population, being more educated and more frequently employed. The most blatant gap related to a family income above the poverty line of 2 USD/day, which was the case for 71% of surrogates and only 24% of women in the general population. This result needs to be confirmed by other empirical studies, as recruitment bias could not be ruled out with a convenience sample. In all field studies, medical doctors or agency managers were necessary intermediaries to recruit surrogates and so it cannot be excluded that the surrogates interviewed may be a selected “presentable” group, leading to possible selection bias.

Our findings showed that the surrogates we interviewed were close to the Indian middle class; this contradicts the common assumption that they are the poorest and least educated women. Some surrogates may be among the disadvantaged Indian population, but this did not appear to be a general rule in our sample. Similar findings were suggested in two ethnographic studies carried out in India. In the first study, the majority of surrogates had previously worked in industry and were not in dire need of income to survive [20]. Surrogates declared that they preferred to be a gestational carrier than to work in industry, where they were underpaid and constantly harassed. The second ethnographic field study concluded that surrogates were not the “*poorest of the poor*” and did not appear as “*powerless victims in need of aid*” [18]. By putting together data from four field surveys, our analysis is the first to provide a quantitative analysis of a convenience sample of 96 Indian surrogates. Our analysis of Indian surrogates is in line with studies in rich countries such as the United States, the UK or Israel, which concluded that surrogates mainly belong to the middle class in their own country [32, 34, 35].

It would have been interesting to explore complementary dimensions of the surrogates’ status, in particular with regard to caste, which is an important sociocultural marker in Indian society. However, this issue is sensitive and was covered only in one field survey. Among the eight surrogates who gave information on their caste [21], five belonged to the Forward Castes (socially advantaged) and three to the Other Backward Castes or the Scheduled Caste (socially disadvantaged). These first limited observations suggest that surrogates may come from both lower and from upper castes.

The relatively favorable social characteristics of surrogates compared with the general population may be due to a selective process in surrogacy arrangements. Firstly, it could be explained by the criteria used by medical doctors to recruit surrogates. They may refuse to contract with women living in slums or on the street because of poor housing, poor hygiene and poor nutrition that could have an adverse impact on the pregnancy. Moreover, medical doctors may recruit women with at least a minimum of social and educational capital, to be sure that they will understand the whole surrogacy process, in particular that the future child will be not genetically related to the surrogate or her husband.

Secondly, on the surrogates’ side, there may be a selective social process. When commercial surrogacy was initiated in 2002 in India, surrogates were recruited by brokers who were generally former surrogates or egg donors, and who recruited “desperate mothers” in their neighborhoods. Then, word of mouth and publicity through social networks appeared to replace brokers, particularly in Chennai where one of the surrogacy agencies ran an advertising campaign in the printed press and on television. Access and integration in the social network is thus probably a factor facilitating the engagement of women in surrogacy. However, 35% of Indian women aged 15–49 are not regularly exposed to any media [28] and could thus be less likely to have been informed about surrogacy.

In addition, before starting a pregnancy and later being placed in a surrogacy home, surrogates must be able to move freely in public space in order to follow the detailed medical protocol, including multiple check-ups at the clinic before embryo transfer. Such journeys to the clinic could be an issue in the daily life of Indian women. Only 48% of women aged 15–49 are allowed to go freely to the medical center, 51% to the market and 40% to travel outside their community [28]. In this gender-related social context, we may postulate that Indian women who have more autonomy and who are better integrated in social networks are more likely to have access to information and to clinics, and so to commit to surrogacy.

Our study did not explore whether surrogacy was a free choice for women. This issue has been investigated in a few field studies which reported no coercion to become surrogates in India. Rudrappa reported that surrogates preferred surrogacy to working in the garment industry [20]. Another aspect concerns the unequal relationship between surrogates and medical doctors, and between surrogates and intended parents from rich countries. Indian surrogates have no power to make decisions about the gestational and birth process, which is decided and managed by medical doctors and intended parents [17, 22]. The Indian situation contrasts with that in the United States, the UK or Israel, where surrogates have autonomy and the power to make decisions on the pregnancy, including on the future child [32, 35, 36].

To conclude, based on a convenience sample, the 96 Indian surrogates interviewed were not among the poorest and least educated women. Further studies would be needed to confirm this first quantitative analysis. In the future, the recruitment of a random sample of surrogates could be considered as the Surrogacy Regulation Bill, approved in December 2018, includes the constitution of a National Surrogacy Board that may make it possible to produce national data on surrogacy.

This Surrogacy Regulation Bill also enforced new guidelines, as surrogacy became local, altruistic and relational, i.e. it is performed altruistically for an Indian married couple by a close female relative. Facing international scandals, this new law aimed to protect the image of India from the degrading perspective of women’s exploitation through surrogacy. Paradoxically, this change could have the perverse effect of promoting a black market and an invisible coercion of women [37, 38]. Moreover, new international destinations have emerged to take the lead in surrogacy, in particular Kenya, Malaysia and Laos [39]. It will be of primary importance to explore who are the new surrogates in these countries where the practice is not yet regulated.

## Supporting information

S1 Appendix Database of the 96 surrogates. (XLSX)
